# Large Foundation Model for Cancer Segmentation

**DOI:** 10.1177/15330338241266205

**Published:** 2024-07-25

**Authors:** Zeyu Ren, Yudong Zhang, Shuihua Wang

**Affiliations:** 1 School of Computing and Mathematical Sciences, 4488 University of Leicester, Leicester, UK; 2 College of Agronomy, Jilin Agricultural University, Changchun, China; 3 Department of Information Technology, Faculty of Computing and Information Technology, King Abdulaziz University, Jeddah, Saudi Arabia; 4 Department of Biological Sciences, Xi’an Jiaotong-Liverpool University, Jiangsu, China

**Keywords:** medical image analysis, image segmentation, deep learning, foundation model, segment anything model (SAM), machine learning

## Abstract

Recently, large language models such as ChatGPT have made huge strides in understanding and generating human-like text and have demonstrated considerable success in natural language processing. These foundation models also perform well in computer vision. However, there is a growing need to use these technologies for specific medical tasks, especially for identifying cancer in images. This paper looks at how these foundation models, such as the segment anything model, could be used for cancer segmentation, discussing the potential benefits and challenges of applying large foundation models to help with cancer diagnoses.

Cancer arises when cells proliferate uncontrollably within a specific part of the body. These malignant cells not only invade but also erode the adjacent tissues and organs, positioning cancer as a leading cause of death globally. In the last few decades, various computer-aided diagnosis systems based on deep learning techniques have been designed to assist doctors in the early detection of cancer.^1^ Among various deep learning applications, medical image segmentation occupies a pivotal role in clinical practice, aiming to enhance accurate diagnosis, treatment planning, and early cancer detection. Nonetheless, the prevailing deep learning techniques always need specific modalities and different types of cancer, which lack generalizability across different modalities, quantities, and types of medical imagery. This limitation complicates the tasks of devising accurate and efficient segmentation models in medical image analysis compared to other domains.

In recent years, the advent of large language models, such as ChatGPT, has demonstrated considerable success and established a significant milestone in the field of natural language processing (NLP). These models pretrained on the web-scale datasets have evolved NLP models by exhibiting strong zero-shot or few-shot generalization capabilities.^2^ With extensive data sourced from the web, these foundation models also show surprising zero and few-shot performance in fine-tuned models.^2^ Recently, the scope of foundation models has expanded into computer vision, the segment anything model (SAM)^3^ a pioneering foundation model for image segmentation, is pretrained on a broad dataset and employs prompt engineering to tackle the proxy segmentation challenges in new data distributions. It requires points or bounding boxes to identify the segmentation targets. Also, the Segment-Everything-Everywhere Model^4^ is proposed, capable of segmenting every element within an image simultaneously. These foundational models have demonstrated exceptional adaptability and efficacy across various segmentation tasks.

While foundational models have demonstrated impressive capabilities in computer vision, there is a growing urgency for their application in medical image segmentation. Recent efforts have seen the applications of SAM in downstream medical segmentation tasks. Some studies directly utilized the SAM to do segmentation, whereas others have employed the out-of-the-box SAM. For instance, Deng et al^5^ access the segmentation performance of SAM directly on cancer segmentation tasks, including the segmentation of the whole-slide images (WSIs) of skin cancer patients, as well as assessing SAM's efficacy in tissue and cell nuclei segmentation. However, it does not consistently achieve a satisfying performance in cancer segmentation. The challenges of directly applying the SAM in medical images can be categorized into 4 areas. Firstly, the resolution of gigapixel WSI cancer data significantly exceeds the average resolution of training images used in SAM, potentially leading to an impractical number of interactions at the patch level. Secondly, digital cancer pathology has multiple scales, and the performance of segmentation models highly relies on the optimal image resolution of digital pathology. However, selecting appropriate scales for cancer images when using the SAM model proves challenging. Moreover, SAM relies on promptable interaction for segmentation, where high-quality prompt selections are essential to achieve superior performance in cancer segmentation. The last challenge is model fine-tuning, the laborious prompt selections are still necessary for cancer segmentation, especially when cancer data have significant domain heterogeneities. Therefore, a reliable strategy for online/offline fine-tuning is essential to transfer the insights gained from manual prompts to broader-scale automatic cancer segmentation.

To address the aforementioned limitations associated with the direct application of SAM for cancer segmentation, numerous studies have adapted the out-of-the-box SAM to medical image segmentation through various strategies. These methodologies include fine-tuning, auto-prompting adaptation, framework modification, and modalities adaptation.^6^ The fine-tuning strategy includes two main types: full fine-tuning and parameter-efficient fine-tuning (PEFT). The full fine-tuning directly uses the target domain's data to fine-tune the SAM. Hu et al^7^ propose the Skinsam, where the SAM specifically fine-tuned for the skin cancer segmentation using the HAM10000 dataset, resulting in a notable improvement in the Dice similarity coefficient score from 82.25% to 88.79%. Another significant contribution is MedSAM,^1^ which aims to bridge the gap between natural images and medical images using foundation models. MedSAM fine-tunes SAM on large-scale medical datasets comprising over 1 million medical images with segmentation masks. It evaluates 86 internal validation tasks and 60 external validation tasks, demonstrating competitive performance against other state-of-the-art foundation models. Nonetheless, MedSAM encounters challenges in accurately delineating vessel-like branching structures, a difficulty attributed to the potential ambiguity in bounding box prompts within these tasks. PEFT represents a strategy where only a subset of SAM's parameters are trained to adapt to specific segmentation tasks, thereby maintaining efficiency. Wu et al^8^ proposed a MedSAM to employ the LoRA module trained alongside a frozen SAM model for segmentation tasks. This approach exemplifies the potential of PEFT techniques in achieving task-specific adaptation without the need for extensive retraining of the entire model.

Auto-prompting adaptation focuses on developing a robust framework to enhance the stability and performance of SAM in medical image segmentation. This adaptation is categorized into 3 main types: prompts generation,^9^ learnable prompts,^10^ and enhancing reliability against prompts with uncertainty.^11^ The quality of standard prompts (such as points, boxes, and masks) decides the effectiveness of medical image segmentation. Therefore, prompts generation tries to generate reliable and accurate prompts for the SAM with an additional localization framework, which tries to generate high-quality prompts with lower noises to enhance the performance of the SAM. Learnable prompts use auxiliary models or strategies to improve the quality of the weak prompts, and then generate more accurate and reliable prompts to improve the performance of the SAM. For example, Gao et al^12^ introduce a decoder to generate mask embeddings based on the existing prompts, then fuse image and mask embeddings to generate final segmentation masks. Shaharabany et al^10^ propose an encoder to produce conditional prompts to the SAM, and then these conditional prompts expand the basic prompts to segment medical images by SAM. Enhancing reliability against prompts with uncertainty suits the clinical practice, which needs higher accuracy. By estimating the uncertainty, it ensures that the final segmentation outcomes are reliable and suitable for medical applications. In detail, uncertainty can guide the model in finding potential errors during the training process, it also offers meaningful corrections to clinicians and improves the segmentation results.

Framework modification involves integrating the SAM with other existing or new-designed structures or models to create a more sophisticated and effective model for medical image segmentation. There are two main directions: enhancing synergy during the training process and enabling efficient annotation learning. The strategy of enhancing synergy during the training process focuses on incorporating additional architectures or models alongside SAM to improve the overall performance of the framework. An example of this is nnSAM proposed by Li et al,^13^ which merges the capabilities of SAM with the nnU-Net pipeline, resulting in higher performance on segmentation tasks. Enabling efficient annotation learning combines weakly supervised learning to reduce the cost of annotation, which is more applicable for the cancer segmentation task when the annotations are limited. For example, SAM can conduct additional segmentation models to select valuable pseudo labels to assist the consistency learning and further refine the segmentation results.

Most of the SAM-based models are focused on 2-dimensional (2D) images in a single modality, the modalities adaptation methods try to segment the medical images across different modalities. It becomes crucial when adapting 2D to 3-dimensional (3D) medical segmentation tasks since 3D segmentation can provide more accurate volume estimation, anatomical details, and spatial contexts. The basic 2D to 3D medical segmentation with the SAM always decreases the performance. Therefore, most of the works present adapters to capture spatial and depth features to the 3D medical segmentation.^8^ In contrast, another way of 3D medical segmentation trains models from scratch with large 3D medical datasets to result in more accurate segmentation outcomes.^14^ Also, there are prospects for further developments in 3D segmentation. Firstly, auto-prompting adaption methods can use additional localization models to generate 3D prompts, or produce 3D embeddings to improve the quality of the existing predictions. Moreover, framework modification-based methods are also suited for 3D segmentation, where extra models can improve the framework's performance.

Although large foundation models such as SAM present great success in segmentation tasks, challenges still exist that need to be solved. Firstly, there are limited large medical datasets with annotations to train foundation models. Even fine-tuning can improve performance, but it is still not sufficient for medical image analysis. Secondly, annotating large medical datasets is important to train the foundation model, especially for 3D segmentation. Therefore, developing a weakly supervised learning-based framework with lower annotation costs is essential. Moreover, there are limited applications of multimodal medical images, where they can provide complementary information on diseases.

Employing foundation models in medical image segmentation also poses ethical implications regarding data privacy, bias mitigation, transparency, and reliability. Hence, protecting patient privacy, addressing biases, ensuring transparency, and validating safety is crucial. Compliance with regulatory standards, respecting patient autonomy through informed consent, and promoting equitable access are also essential. Additionally, considering the long-term impact on healthcare professionals’ roles is necessary for responsible deployment, and collaboration among stakeholders is vital to navigate these complexities and ensure the ethical use of artificial intelligence in healthcare.

Over the last few years, large foundation models have shown unprecedented potential in many domains, especially in different medical segmentation tasks. Introducing large foundation models for cancer segmentation is crucial to get promising treatment planning, and clinical practice results, and many potentials need to be explored by using a large foundation model for cancer segmentation in the future.

## Abbreviations

NLP: natural language processing
PEFT: parameter-efficient fine-tuning
SAM: segment anything model
WSI: whole-slide image.
